# Transcriptomic and biochemical analyses of drought response mechanism in mung bean (*Vignaradiata* (L.) Wilczek) leaves

**DOI:** 10.1371/journal.pone.0285400

**Published:** 2023-05-10

**Authors:** Yaning Guo, Siyu Zhang, Jing Ai, Panpan Zhang, Han Yao, Yunfei Liu, Xiong Zhang

**Affiliations:** College of Life Science, Yulin University, Yulin, Shannxi Province, China; Jeju National University, REPUBLIC OF KOREA

## Abstract

Drought is a major factor that limiting mung bean development. To clarify the molecular mechanism of mung bean in response to drought stress, 2 mung bean groups were established, the experimental group (drought-treated) and the control group (normal water management). With prominent difference of 2 groups in stomatal conductance, relative water content and phenotype, leaf samples were collected at 4 stages, and the physiological index of MDA, POD, chlorophyll, and soluble proteins were estimated. RNA-seq was used to obtain high quality data of samples, and differentially expressed genes were identified by DESeq2. With GO and KEGG analysis, DEGs were enriched into different classifications and pathways. WGCNA was used to detect the relationship between physiological traits and genes, and qPCR was performed to confirm the accuracy of the data. We obtained 169.49 Gb of clean data from 24 samples, and the Q30 of each date all exceeded 94%. In total, 8963 DEGs were identified at 4 stages between the control and treated samples, and the DEGs were involved in most biological processes. 1270 TFs screened from DEGs were clustered into 158 TF families, such as AP2, RLK-Pelle-DLSVA, and NAC TF families. Genes related to physiological traits were closely related to plant hormone signaling, carotenoid biosynthesis, chlorophyll metabolism, and protein processing. This paper provides a large amount of data for drought research in mung bean.

## Introduction

Drought is one of the most important abiotic stresses that limits plant growth and productivity seriously [1]. In the face of continuous water deficits, plants respond with several changes at the molecular, biochemical, physiological, and morphological levels [2]. At the molecular level, a series of genes change their expression level. Such as genes involved in abscisic acid (ABA) metabolism play an important role in response to water deficit stress by regulating stomatal movements [3,4]. At the biochemical and physiological levels, antioxidant protection systems perform an essential function. As the product of peroxidation, malondialdehyde content (MDA) reflects membrane degradation in plants, and MDA concentrations is connected to plant tolerance to water deficit stress [5]. Peroxidase (POD) plays a key role in drought tolerance of mung bean [6]. At the morphological level, transpiration weakened firstly to reduce water evaporation, after which the relative water content decreased in the leaves [7]. As water deficits increased, wilting appeared, and the height and color of plants changed [8].

Mung bean (*Vigna radiata* L.) is an important legume crop in Asia with high stress resistance and short growing period [9]. Due to its high protein content in seeds and sprouts, mung bean has become more popular, and the planting area has increased in recent years [10]. Mung bean is widely cultivated in dry regions, as it is highly resistant to drought conditions [11]. Transcriptome analysis is typically used to reveal gene networks, and transcriptome analysis combined with physiological and biochemical indicators can help identify candidate genes for drought tolerance [12–14].

The increased availability of mung bean genome sequences resulted in more conveniently mung bean genomic analysis [15–17]. To explore the inner mechanism of drought resistance in mung bean, leaf samples at 4 stages were obtained from control and drought-treated mung bean, also the morphological and physiological changes were detected in this study. Based on RNA-seq, DEGs were analyzed to clarify the molecular mechanism of mung bean in response to drought stress. The relationship between molecular and physiological changes was analyzed to screen candidate gene response to drought stress in mung bean, which provides a vital foundation for future studies assessing drought resistance in mung bean.

## Materials and methods

### Sample collection and preparation

The mung bean cultivar Yulv 1 was used for drought treatment and leaf samples were obtained to perform RNA-seq. Healthy and uniform seeds were selected and cultivated in a pot under an arain shelter, with a cycle of 16 h light and 8 h dark, and temperature in 23°C-29°C. The soil composition and weight were the same, and the soil water content was maintained at a relative humidity of 70%-80%.

An experimental group (T) and a control group (C) were set, each group containing 100 pots, and every pot growing three plantlets. After 15 days of sowing, when the first ternate leaf fully expanded, drought treatment was performed. The experimental group without water, and the control group received normal water management. The parietal leaves of the first ternate leaf were sampled from 4 stages. The first stage was determined according to the stomatal conductance data between two groups (G0). Significant difference of the stomatal conductance between the 2 groups appeared after 3 days of treatment, and the experimental group reduced to 0.16 mol H_2_O m^-2^s^-1^, while the control group was 0.24 mol H_2_O m^-2^s^-1^. The second stage was determined based on the leaf relative water content (G1). After 6 days of treatment, relative water content of the first ternate leaf between the experimental group (89%) and control group (68%) appeared significant difference. After 9 days of no irrigation, the third stage began when the leaves of the experimental group began wilting (G2), and the final stage began when the experimental group was rehydrated for 24 hours (G3). All samples were maintained in liquid nitrogen for subsequent use. Each sample contained three biological repetitions, and related data for G0 and G1 shown in S1 Appendix.

### Morphological and physiological index measurements

The stomatal conductance and other photosynthesis indices of the leaf samples were detected by LI-6400 (LI-COR, USA) [18]. The relative water content was determined as previously described [19]. The MDA content, soluble protein content, and chlorophyll content were measured based on the method described by Guo [20]. POD activity was detected according to the method described by Kong [21].

### RNA preparation and sequence assembly

RNA was extracted using a plant RNA purification kit (TianGen, Beijing). Quality was confirmed using values of 260/280≥1.8, 260/230≥2.0, 28S/18S≥1.8, and RIN≥7.5, and gel electrophoresis was used to ensure RNA integrity. Each sample contained three biological repetitions. More than 2 μg of RNA of each sample was used for sequencing on Illumina 2500 sequencing system. All data were deposited in CNCB-NGDC SRA (https://ngdc.cncb.ac.cn/databases) under the accession number CRA008526.

### Identification of differentially expressed genes

Raw RNA-Seq reads were filtered, and low-quality reads containing ploy-N whose unknown bases percentage exceeded 10% were removed. Q30 and GC-content were checked to ensure the quality of clean reads. With HISAT, the clean reads were matched to the genomic data (ftp://ftp.ncbi.nlm.nih.gov/genomes/all/GCF/000/741/045/GCF_000741045.1_Vradiata_ver6) [22,23], and StringTie was used to assemble the reads [24]. DEGs were identified by DESeq2 software with a fold change≥1.5 between different samples and P-value≤0.05 [25]. Three biological repetitions were performed, and Pearson’s correlation coefficient (r) was calculated to confirm the DEGs.

### qRT-PCR analysis

Total RNA was extracted with an RNAprep Pure Kit DP441 (Tiangen, Beijing). Extracted RNA quality and concentration were contained by NanoDrop and 1% agarose gel electrophoresis, with 260/280≥1.8 and 260/230≥2.0 [26]. cDNA was synthesized by strictly following the protocol of TransScript^®^One-Step Gdna Removal and Cdna Synthesis SuperMix (Transgen, Beijing). qPCR was conducted with *TransStart*^®^ Top Green qPCR SuperMix (Transgen, Beijing) on an ABI7500 instrument (Applied Biosystems, United States) using *Vractins* as the reference gene [27]. Primers of selected genes for qPCR were designed by Oligo 7, and the specificity of each primer was guaranteed by melting peaks and dissociation curves (Table 1). The resulting data was calculated by the 2^-(ΔΔCT)^ method [28], for Δ_1_ = CT(gene)—CT(actins), and Δ_2_ = Δ_1_(Treatment)—Δ_1_(Control). Statistical significance was calculated using *t*-tests with SPSS software [29]. All data were obtained in triplicate.

**Table 1:** Primers for qRT-PCR.

Name	Forward primer (50 → 30)	Reverse primer (50 → 30)	product size (bp)
*gene11285*	GCACGAGTATCGCCTCCTTGA	CGAACCGTTGCTGTATTGAGTG	160
*gene29041*	GCAAGTTTCAACCTTCCTCCTG	CACCTATTTCCTTCCAACACCC	244
*gene14347*	TCTGGGATAATGAGGTTGGA	TCGTTGAGCGGTTTAGTGTT	107
*gene23332*	ATCTCATTTCAAGGCAGTCTCAG	CAACTCAAGTTATCCGCTTCTAT	82
*gene9387*	TGTGGGAGGGAAAGAGTGGT	CCTTGCGTAGGATAGGTCTGT	131
*gene1255*	CATTACCTGCTTCAGTCATTCC	CTTCATTGCTATCACAAACCCT	189
*gene13030*	CCACGAGACAGAAAGTACCCAAACG	GATCGCCTTGTCAGTGCCAGTG	90
*gene14215*	TTATGGAGCACTGGGACTTG	TACCCTTTGAGTTTGGACAGA	119
*gene22209*	GTTCCTCTTCCTGTCGCCATCATC	AAGTACCACTCTTGCTCGCCAAAC	101
*Vractins*	GTCGCACCACCAGAGAGGAAATAC	ATACTCAGCCTTCGCAATCCACATC	99

## Results

### Morphological and physiological changes of mung bean leaves under drought stress

**Fig 1:**
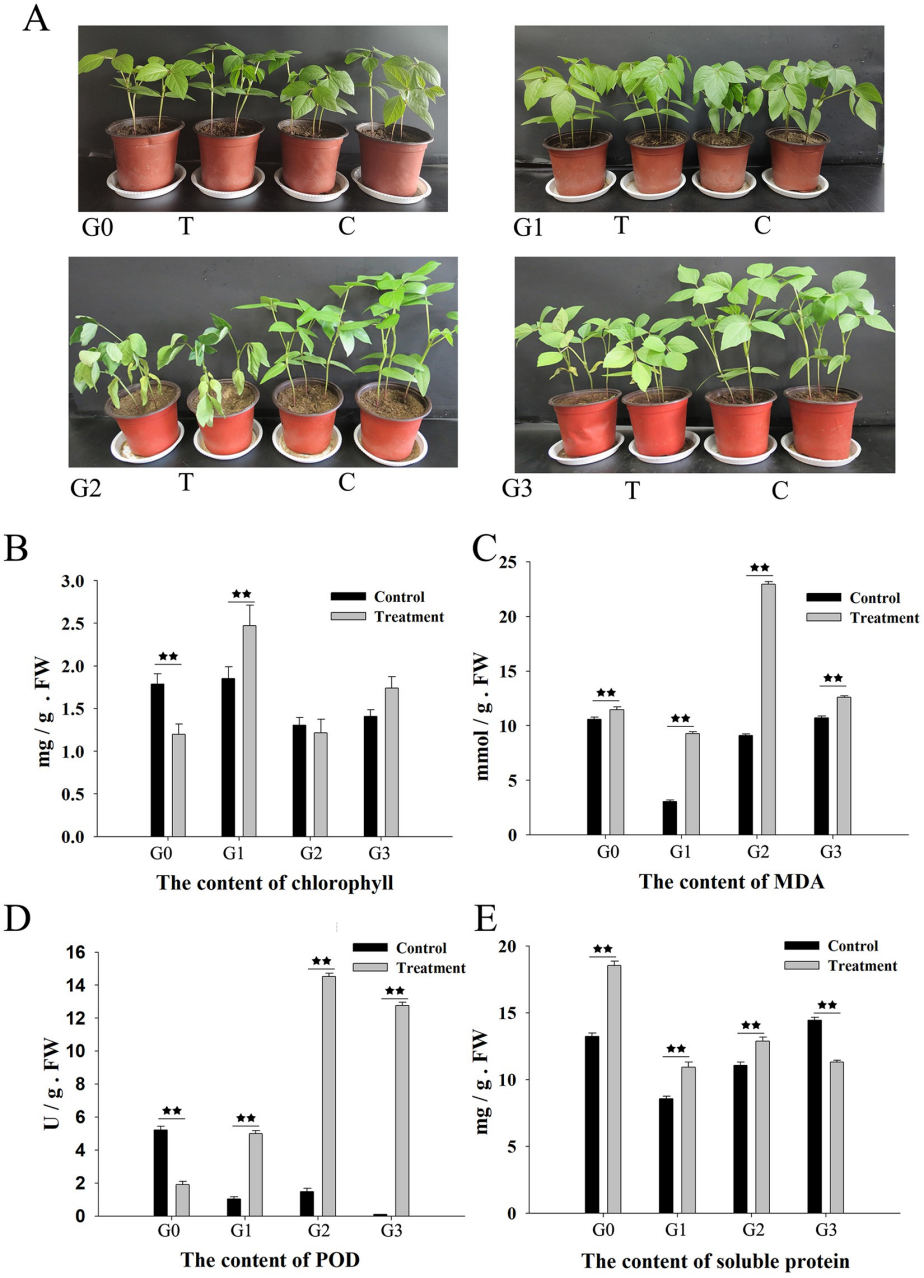
Physiological changes of mung bean under drought stress. A: Phenotype of water deficit treated mung bean at 4 stages. G0 for stomatal conductance, 2.44 mol H_2_O m^-2^s^-1^ in C and 0.16 mol H_2_O m^-2^s^-1^ in T. G1 for relative water content, 89% in C and 68% in T. G2 for obvious wilting phenotype in T, and G3 was rehydration for 24 hour in T. C: Control, T: Treatment. B: The chlorophyll content in different samples. C: The MDA content. D: The POD activity. E: The soluble protein content. Data are shown as the mean ± SD (*n* = 3). ** represent extremely significant difference between control and treatment samples (P<0.01).

To explore the morphological and physiological changes of mung bean under drought stress, the content of chlorophyll, MDA, soluble protein and POD activity were determined at the 4 stages (Fig 1). The drought-treated mung bean displayed stunted growth (Fig 1A). As shown in Fig 1B, the chlorophyll contents of leaf samples were significantly different between the control and the treatment samples at G0 and G1 stage, and the treated sample had higher chlorophyll contents at G1. There was no statistically significant difference at G2 and G3 stages, which meant that drought stress initially promoted chlorophyll accumulation. In Fig 1C, the MDA content accumulated in drought-treated samples, and once the leaves wilted, the drought-treated sample had the highest MDA content in the leaves. This indicated that MDA was induced by water stress. In Fig 1D, the POD content was significantly different between the control and treated samples at the 4 stages, and peaked at the G2 stage. In Fig 1E, the soluble protein content increased during the first stage, and decreased in the G1 and G2 stages. However, at G3 stage, the soluble protein content in the control group was significantly higher than that in the treated group.

### High-throughput of RNA-Seq

**Fig 2:**
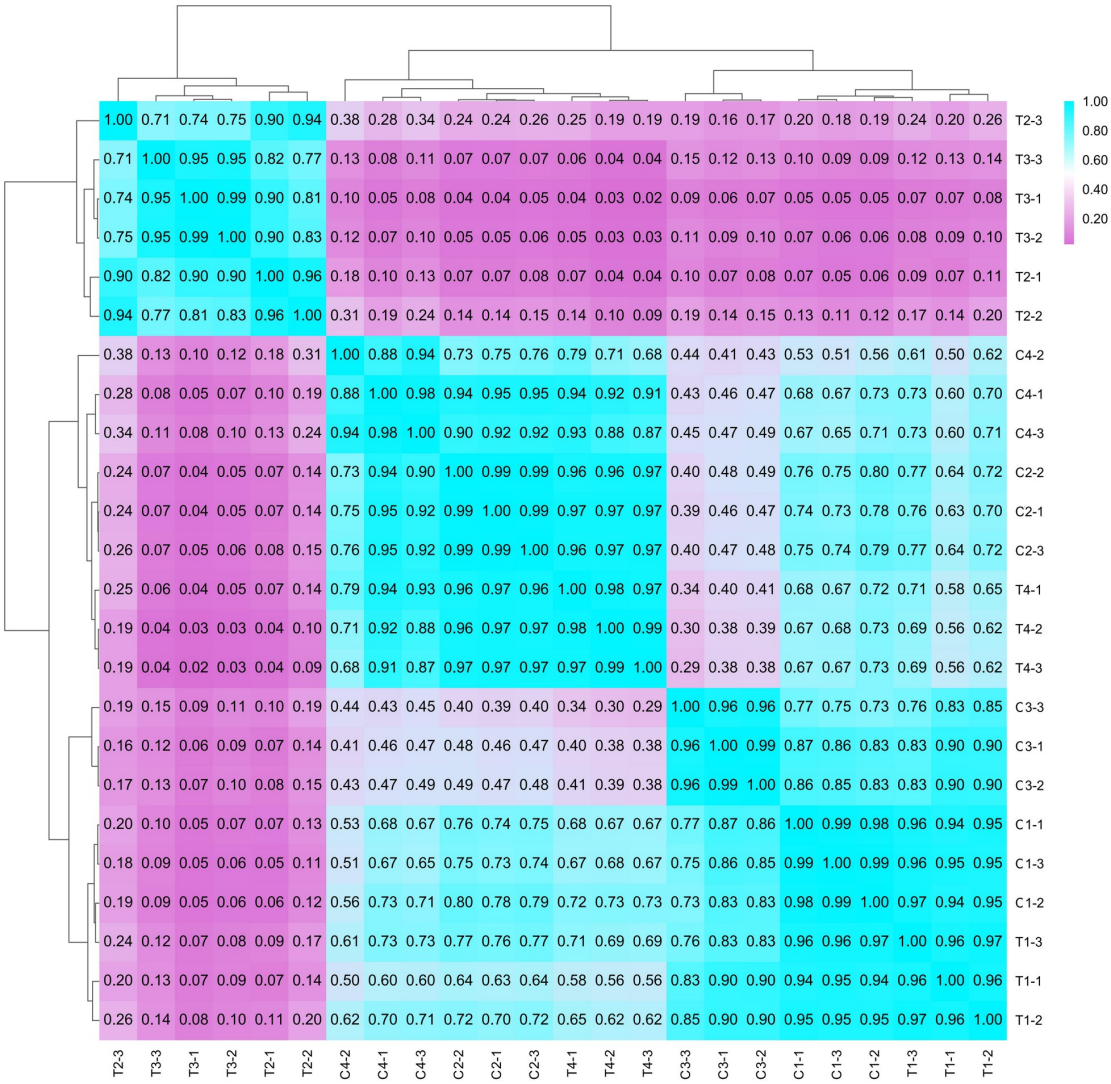
The correlation coefficient between different samples. Numbers represented correlation coefficient. C1-C4 represented samples of the control group at 4 stages. T1-T4 represented samples of the treated group at 4 stages. Each sample contained 3 repetitions.

After sequencing, clean data was obtained by assessing the quality score, base distribution, and Q30 value, and filtering low-quality sequences, finally clean data was obtained. Based on the genome database of mung bean [22], clean data was assembled by the HISAT2 system and String Tie algorithm [24]. To ensure accuracy, three biological repetitions were included, and the r value was calculated in Fig 2. The correlation coefficient in different repetitions for one sample was close to 1, where T2 had a high correlation coefficient with T3. This indicated that the expression of genes at the G1 and G2 stages had a relatively high consistency. The rates of clean reads paired to chromosomes all exceeded 87%, and clean reads were evenly distributed on all 11 chromosomes of mung bean, indicating the reliability of RNA-Seq data (S2 Appendix).

### DEGs between drought conditions and control at 4 stages

**Fig 3:**
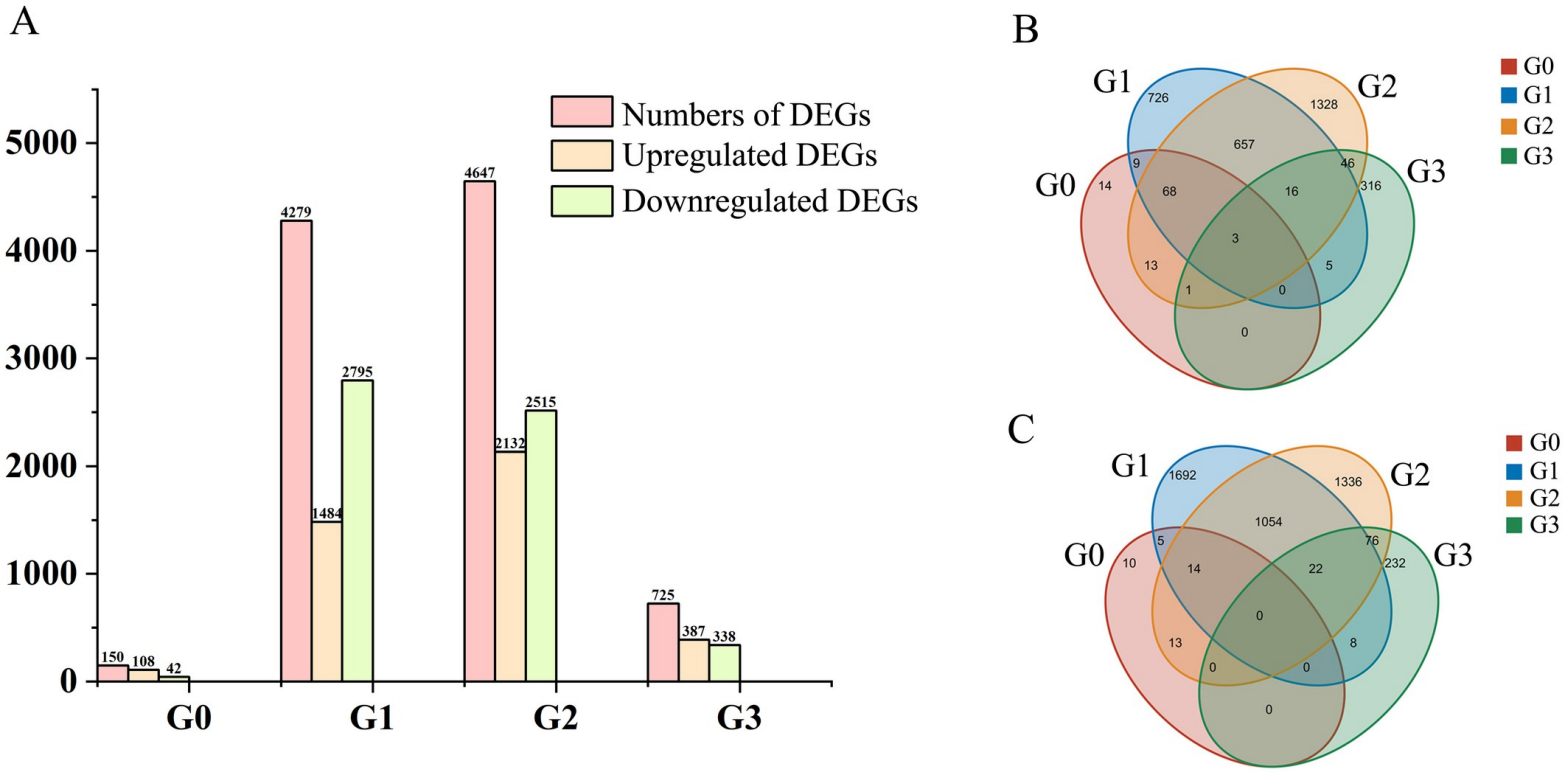
DGEs in different leaf samples. A: The numbers of DEGs in different samples. B: The venn diagram of up-regulated genes in samples. C: The venn diagram of down-regulated genes in samples. G0: C1-T1, G1: C2-T2, G2: C3-T3, G3: C4-T4, for C1-C4 and T1-T4, three biological repetitions were included in each sample.

With DESeq2_EBSeq, the parameter was set as |log2(Fold Change)| >1 and FDR < 0.01, and 8963 DEGs were identified between the control and treatment samples from 4 stages (Fig 3). Of these, 150 DEGs were in G0, 4279 DEGs in G0, 4647 DEGs in G2, and 625 DEGs were in G3 stage. There were more DEGs at G1 and G2, and after 24 hours of rehydration, DEGs in the control and treated leaves decreased. For up-regulated genes, there were 3 genes up-regulated in all of the 4 stages. 80 genes up-regulated both at the G0 and G1, 744 genes up-regulated both at the G1 and G2, 70 genes up-regulated both at the G2 and G3, and 4 co-genes up-regulated both at the G0 and G3. For down-regulated genes, 19 down-regulated genes both at G0 and G1, 1103 co-genes down-regulated both at G1 and G2, and 98 genes down-regulated both at G2 and G3. No co-genes decreased at the G0 and G3. With drought prolonged, the amount of DEGs increased from G0 to G2, while after 24 h of rehydration (G3), the number of DEGs decreased to 725. This meant that many genes in mung bean were involved in drought response.

### GO enrichment in DEGs

To identify the major biological processes involved in drought response, GO enrichment analysis was performed with FDR < 0.01 and FC = 2. DEGs in both the control and the drought-treated samples at different stages were enriched into various biological processes (S3 Appendix). The simplified GO enrichment results for DEGs were shown in Table 2. At G0 stage, genes mainly enriched in metabolism of membrane and cytoplasmic metabolism. At G1 stage, both of the up-regulated and down-regulated genes mainly enriched in the functions of biological regulation, metabolic process, cellular process, nucleus, cytoplasm, organelle, membrane, protein binding, metal ion binding and ATP binding, and the down-regulated genes additionally enriched in functions of cell wall organization, response to stimulus, protein kinase activity, hydrolase activity, oxidoreductase activity, catalytic activity and molecular function. At G2 stage, regulation of transcription, biological process, cell, organelle, nucleus, transcription factor activity, metal ion binding, DNA binding, protein binding, catalytic activity, membrane, ATP binding and molecular function were enriched both in the down-regulated and up-regulated genes. In the same time, functions in response to stimulus, metabolic process, hydrolase activity and zinc ion binding were specially enriched in up-regulated genes, and down-regulated genes also enriched in carbohydrate metabolic process, cellular process, biological process, protein kinase activity, oxidoreductase activity and protein serine/threonine kinase activity. At the G3 stage, the number of DEGs between the control and treated samples decreased, and these genes were mainly involved in ATP binding and membrane function. From G0 to G3 stage, drought stress gradually became serious. At the initiation of drought stress, genes were enriched in cell permeability regulation, which could help mung bean relieving drought pressure. With drought stress sustaining, plants enhanced the response to stimulus. Organics substance hydrolyzed to increase cell osmotic potential, and oxidoreductase reduced the damage produced by peroxidation, then cell wall changed, carbon metabolism strengthened, and finally cell permeability regulation was enhanced, which guaranteed plant survival from serious drought stress.

**Table 2:** Statistics of Gene Ontology analysis for DEGs at 4 stages.

Stage	Up-regulated				Down-regulated			
	GO	Description	P	DEGs	GO	Description	P	DEGs
	accession			number	accession			number
G1	GO:0065007	Biological regulation	0.23	51	GO:0071555	Cell wall organization	0.00	51
	GO:0008150	Biological process	0.23	106	GO:0005975	Carbohydrate metabolic process	0.00	71
	GO:0008152	Metabolic process	0.53	52	GO:0050896	Response to stimulus	0.62	70
	GO:0044237	Cellular metabolic process	0.57	48	GO:0008152	Metabolic process	0.95	52
	GO:0009987	Cellular process	0.58	69	GO:0050789	Regulation of biological process	0.95	55
	GO:0005634	Nucleus	0.00	174	GO:0008150	Biological process	0.95	148
	GO:0005575	Cellular component	0.05	107	GO:0009987	Cellular process	0.95	105
	GO:0005623	cell	0.05	105	GO:0065007	Biological regulation	0.95	56
	GO:0044464	Cell part	0.05	105	GO:0016021	Integral component of membrane	0.00	798
	GO:0005622	Intracellular	0.07	97	GO:0005618	Cell wall	0.00	50
	GO:0044424	Intracellular part	0.08	96	GO:0005576	Extracellular region	0.00	57
	GO:0005737	Cytoplasm	0.13	75	GO:0071944	Cell periphery	0.00	55
	GO:0044444	Cytoplasmic part	0.14	50	GO:0009507	Chloroplast	0.00	68
	GO:0043226	Organelle	0.28	76	GO:0016020	Membrane	0.01	83
	GO:0043229	Intracellular organelle	0.30	75	GO:0005886	Plasma membrane	0.02	64
	GO:0043227	Membrane bounded organelle	0.38	70	GO:0044444	Cytoplasmic part	0.78	87
	GO:0016021	Integral component of membrane	0.71	270	GO:0005737	Cytoplasm	0.81	122
	GO:0003677	DNA binding	0.00	109	GO:0005575	Cellular component	0.81	154
	GO:0003674	Molecular function	0.48	74	GO:0005623	Cell	0.81	150
	GO:0005515	Protein binding	0.48	53	GO:0044464	Cell part	0.81	150
	GO:0046872	Metal ion binding	0.58	61	GO:0043227	Membrane bounded organelle	0.81	103
	GO:0005524	ATP binding	0.61	126	GO:0043226	Organelle	0.81	108
	GO:0006355	Regulation of transcription	0.23	63	GO:0004553	Hydrolase activity	0.00	50
	GO:0043231	Intracellular membrane bounded organelle	0.42	68	GO:0043231	Intracellular membrane bounded organelle	0.81	102
	GO:0003700	Transcription factor activity	0.05	59	GO:0005622	Intracellular	0.81	121
					GO:0022857	Transmembrane transporter activity	0.05	52
					GO:0005634	Nucleus	0.81	118
					GO:0004672	Protein kinase activity	0.00	128
					GO:0016491	Oxidoreductase activity	0.03	64
					GO:0005524	ATP binding	0.00	295
					GO:0043229	Intracellular organelle	0.81	107
					GO:0044424	Intracellular part	0.81	121
					GO:0020037	Heme binding	0.20	56
					GO:0005515	Protein binding	0.88	78
					GO:0016787	Hydrolase activity	0.88	61
					GO:0046872	Metal ion binding	0.88	94
					GO:0005488	Binding	0.88	70
					GO:0003824	Catalytic activity	0.88	73
					GO:0003674	Molecular function	0.88	100
					GO:0003677	DNA binding	0.88	60
G2	GO:0009987	Cellular process	0.99	73	GO:0005975	Carbohydrate metabolic process	0.04	56
	GO:0050896	Response to stimulus	0.41	56	GO:0050794	Regulation of cellular process	0.76	51
	GO:0006355	Regulation of transcription	0.86	63	GO:0022857	Trans-membrane transporter activity	0.00	62
	GO:0008150	Biological process	0.92	118	GO:0065007	Biological regulation	0.89	57
	GO:0008152	Metabolic process	0.99	51	GO:0008150	Biological process	0.96	123
	GO:0016021	Integral component of membrane	0.01	460	GO:0003677	DNA binding	0.91	106
	GO:0044444	Cytoplasmic part	0.41	70	GO:0050789	Regulation of biological process	0.80	56
	GO:0005737	Cytoplasm	0.41	103	GO:0009987	Cellular process	0.96	85
	GO:0005623	Cell	0.59	129	GO:0016021	Integral component of membrane	0.00	735
	GO:0044464	Cell part	0.59	129	GO:0005886	Plasma membrane	0.16	56
	GO:0005575	Cellular component	0.64	129	GO:0016020	Membrane	0.61	60
	GO:0044424	Intracellular part	0.69	115	GO:0044444	Cytoplasmic part	0.94	57
	GO:0005622	Intracellular	0.69	115	GO:0005737	cytoplasm	0.94	89
	GO:0043226	Organelle	0.80	97	GO:0005623	cell	0.94	122
	GO:0043227	Membrane bounded organelle	0.80	93	GO:0044464	Cell part	0.94	122
	GO:0043229	Intracellular organelle	0.83	95	GO:0005575	Cellular component	0.94	123
	GO:0043231	Intracellular membrane bounded organelle	0.85	90	GO:0043231	Intracellular membrane bounded organelle	0.94	83
	GO:0005634	Nucleus	0.89	164	GO:0043226	organelle	0.94	89
	GO:0003700	Transcription factor activity	0.32	64	GO:0004674	Protein serine/ threonine kinase activity	0.37	51
	GO:0016787	Hydrolase activity	0.50	60	GO:0005634	nucleus	0.94	180
	GO:0046872	Metal ion binding	0.56	87	GO:0043229	Intracellular organelle	0.94	88
	GO:0003677	DNA binding	0.61	103	GO:0044424	Intracellular part	0.94	103
	GO:0005515	Protein binding	0.80	64	GO:0005622	Intracellular	0.94	103
	GO:0003824	Catalytic activity	0.87	62	GO:0004672	Protein kinase activity	0.00	124
	GO:0005524	ATP binding	0.89	160	GO:0043227	Membrane bounded organelle	0.94	85
	GO:0003674	Molecular function	0.92	81	GO:0005524	ATP binding	0.00	284
	GO:0005488	Binding	0.92	51	GO:0016491	Oxidoreductase activity	0.08	57
	GO:0008270	Zinc ion binding	0.92	50	GO:0005515	Protein binding	0.91	71
					GO:0003700	Transcription factor activity	0.55	72
					GO:0006355	Regulation of transcription	0.96	65
					GO:0046872	Metal ion binding	0.91	83
					GO:0005488	Binding	0.91	64
					GO:0003674	Molecular function	0.91	90
					GO:0003824	Catalytic activity	0.91	56
G3	GO:0016021	Integral component of membrane	0.12	100	GO:0016021	Integral component of membrane	0.35	83
	GO:0005524	ATP binding	0.00	62				

DEGs were identified with |log^2^(Fold Change)| >1 and FDR < 0.01. G0: C1-T1, G1: C2-T2, G2: C3-T3, G3: C4-T4, for C1-C4 and T1-T4, three biological repetitions were included in each sample.

### KEGG pathway enrichment analysis

A total of 131 KEGG pathways were mapped (S4 Appendix), and the significantly enriched KEGG pathways are shown in Table 3. During the initial stages of drought treatment, up-regulated genes were mainly involved in plant hormone signal transduction and the protein processing pathway, and down-regulated genes were enriched in the MAPK signaling pathway. As drought stress continued, genes related to plant hormone signal transduction were enriched, while DEGs related to plant pathogen interactions, carbon metabolism, starch and sucrose metabolism, and carbon metabolism became active. When the leaves of treated mung bean began wilting, 102 DEGs in plant hormone signal transduction were down-regulated, and 33 DEGs were up-regulated. DEGs related to plant-pathogen interactions were enriched, while 131 DEGs were down-regulated and 49 DEGs were up-regulated. The starch and sucrose metabolism and MAPK signaling pathways were enriched with DEGs, either down-regulated or up-regulated. After 24 h of rehydration, the number of DEGs between control and treated samples decreased, and DEGs mainly enriched in the pathways of plant pathogen interactions, phenylpropanoid biosynthesis, and starch and sucrose metabolism.

**Table 3:** Visualization of the enriched biological processes for KEGG analysis.

KEGG pathway	Up-regulated				Down-regulated			
	G0	G1	G2	G3	G0	G1	G2	G3
ABC transporters	0	13	17	6	0	17	25	4
MAPK signaling pathway	2	47	33	12	5	65	60	5
Phosphatidylinositol signaling system	1	5	10	1	0	8	11	0
Plant hormone signal transduction	5	29	59	14	3	72	102	5
Ubiquitin mediated proteolysis	2	15	23	7	0	11	8	2
Basal transcription factors	0	24	7	0	0	3	2	1
Protein processing in endoplasmic reticulum	5	16	51	3	0	41	12	3
Spliceosome	0	13	28	4	0	17	11	2
Plant-pathogen interaction	2	26	49	21	1	114	131	6
Phagosome	0	1	7	5	0	25	11	0
Endocytosis	0	10	12	2	1	33	23	1
Amino sugar and nucleotide sugar metabolism	0	12	17	6	1	46	27	4
Biosynthesis of amino acids	0	12	25	8	1	11	38	2
Carbon fixation in photosynthetic organisms	1	5	5	1	0	26	22	0
Carbon metabolism	1	24	33	6	1	59	52	2
Cutin, suberine and wax biosynthesis	2	9	15	2	0	6	3	1
Cyanoamino acid metabolism	0	4	4	2	0	16	14	5
Fructose and mannose metabolism	0	0	3	2	0	22	19	2
Galactose metabolism	3	8	19	2	0	21	24	5
Glycerolipid metabolism	1	14	24	1	0	19	13	1
Glycine, serine and threonine metabolism	0	4	6	2	1	23	15	2
Glycolysis / Gluconeogenesis	0	15	26	3	0	23	20	1
Glyoxylate and dicarboxylate metabolism	0	8	10	1	0	29	24	0
Inositol phosphate metabolism	3	7	13	0	0	11	14	2
Pentose and glucuronate interconversions	0	8	14	4	0	36	21	1
Phenylpropanoid biosynthesis	1	11	23	11	1	35	34	8
Photosynthesis	0	1	0	1	0	28	30	0
Purine metabolism	0	15	22	6	0	21	10	1
Starch and sucrose metabolism	1	22	33	10	0	67	65	10
Valine, leucine and isoleucine metabolism	2	19	32	2	0	0	0	0

Significantly enriched KEGG pathways for DEGs between control and treated samples in mung bean, with a q-value≤0.05. Three biological repetitions included.

### Identification of putative TFs

**Fig 4:**
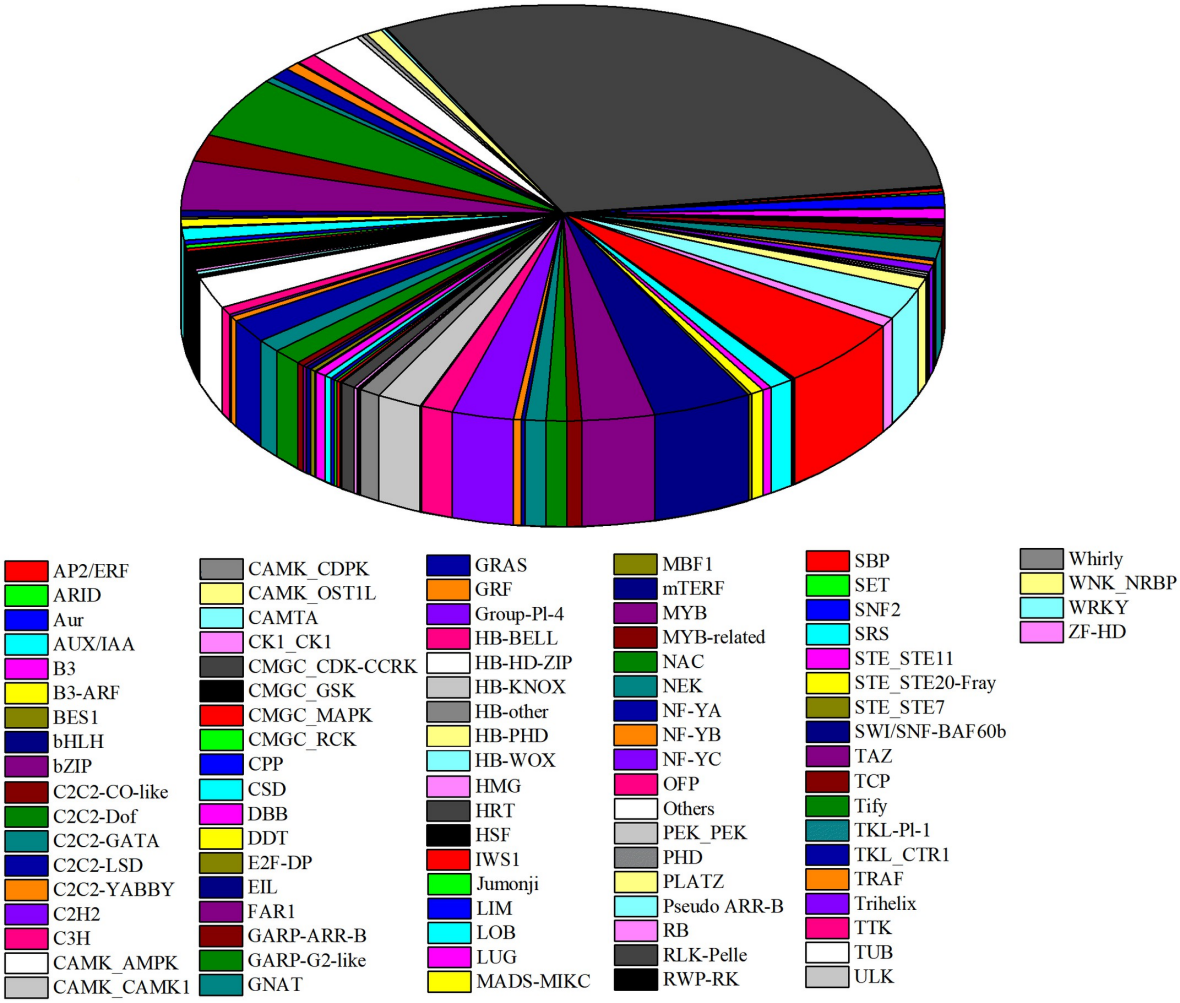
Analysis of TFs in DEGs. Different colors represented various TF families identified from DEGs in 4 stages.

Transcription factors (TF) play important roles in plant development and stress tolerance [30]. In this study, 1270 TFs coming from 158 TF families were screened from total DEGs (S5 Appendix), visualization shown in Fig 4. Members of the RLK-Pelle-DLSV family made up 6% of the total TFs, which was reported to regulate plant growth and interactions with pathogens [31]. AP2/ERF-ERF TF plays an important role in salt and drought stress response in plants [32,33]. In this study, after drought stress, the expression level of AP2/ERF varied, and the percentage was 5%. NAC transcription factors are known to regulate plant response to environmental stress [34]. During the early stages of drought stress in mung bean, 3 NAC members were quickly up-regulated. The TF families of bHLH and MYB comprised 4% of the total TFs, bZIP, RLK_pelle_LRR-X1-1, C2H2, HB-HD-ZIP, and WRKY comprising 3% of the total TFs, and all others comprised less than 3%.

### Gene co-expression network analysis and functional enrichment analysis

**Fig 5:**
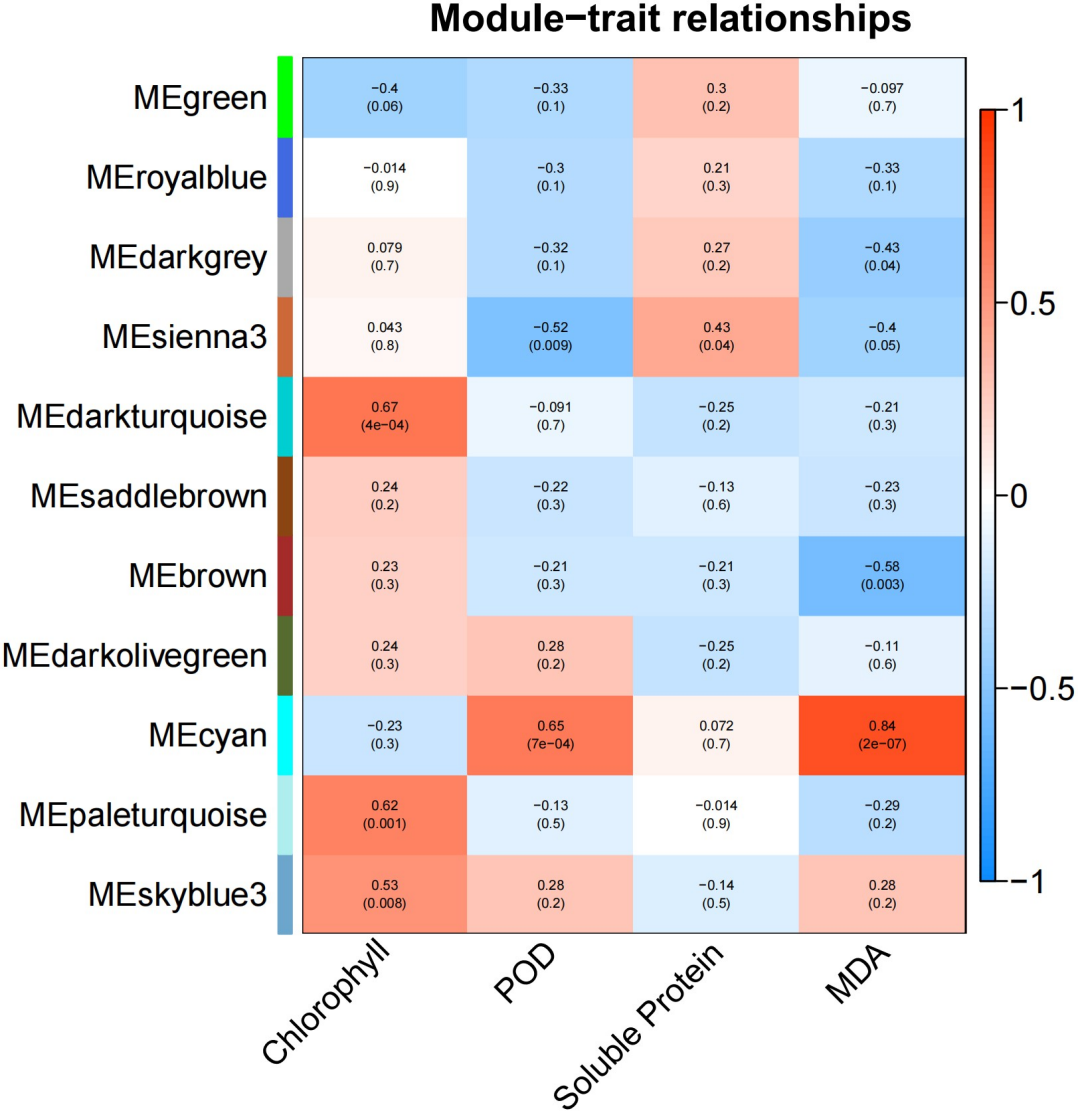
Module-trait relationship with physiological traits. The number indicated the correlation coefficient in modules with physiological traits. The number in the bracket was the *p-value*.

To detect the relationship between physiological traits and gene co-expression in the control and the drought-treated leaf samples, weighted gene co-expression network analysis (WGCNA) was used, parameters set with an expression threshold above 1 and a module similarity threshold of 0.25, with a minimum number of 30 genes in each module. A total of 11 co-expression modules and correlation coefficients were obtained (Fig 5). The module with correlation coefficient above 0.60 and p <0.05 was defined as the physiological indicator-specific module. Chlorophyll content was positively associated with the darkturquoise module (0.67) and paleturquoise module (0.62), and the two modules were primarily enriched in zeatin biosynthesis, circadian rhythm, MAPK signaling pathway, plant hormone signal transduction and fatty acid metabolism (S6 Appendix), which inferred that plant hormone participated in regulation of chlorophyll content in mung bean. POD activity and MDA content both had a positive relationship with the cyan module, and related genes were mainly involved in protein processing in endoplasmic reticulum and circadian rhythm, which indicated that POD activity and MDA metabolism influenced by protein processing.

### Confirmation of RNA-seq sequencing data by qRT-PCR analysis

**Fig 6:**
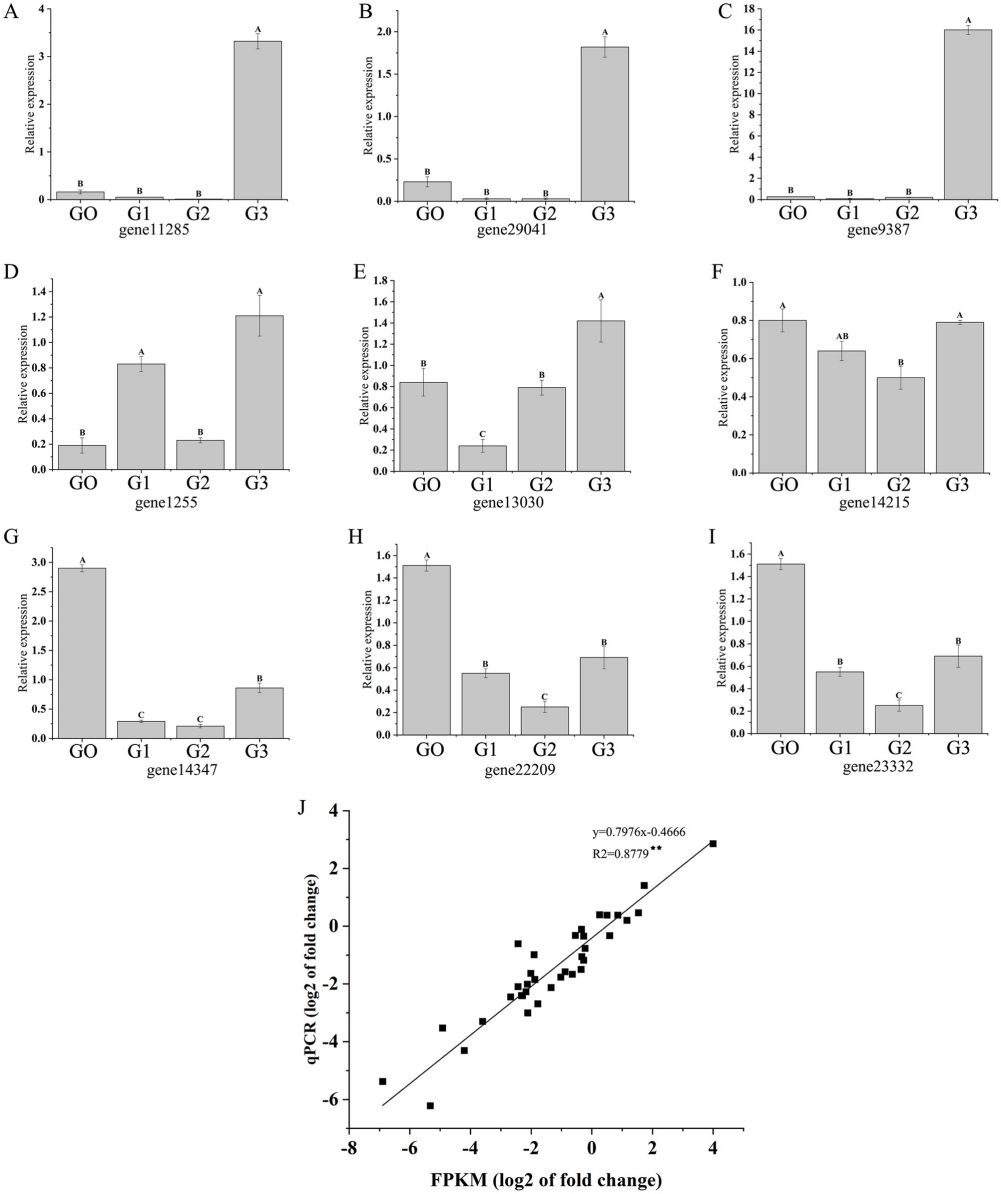
The expression level of selected genes in different stages. A-I: Relative expression of 9 genes. G0-G3 indicated 4 stages of drought stress treatment. J: Comparison between the relative expressions obtained from qPCR and RNA-Seq. X-axis was the log^2^ value of relative expression. Y-axis was the log^2^ value of FPKM in RNA-Seq. Data are shown as the mean ± SD (*n* = 3). Different letters in each figure indicated significant difference at 0.01 level.

To confirm the accuracy of the RNA-seq sequencing, the relative expression of 9 genes was detected by qPCR (Fig 6). The relative expression trend of the selected genes detected by qPCR was positively correlated with RNA-seq sequencing data, which confirmed the accuracy of the RNA-seq results. NAC transcription factor play important roles in mung bean response to drought stress [35]. Expression of the 9 selected NAC genes showed significant difference. For gene11285, gene29041, gene1255, gene9387 and gene14347, they presented extremely significantly up-regulation at G3, while for gene22209 and gene23332, they showed the lowest relative expression at G2, and the highest relative expression at G0. The expression of the other 2 genes also exhibited significant difference at different stages, which indicated that NAC also played a crucial role in mung bean response to drought stress.

## Discussion

Drought is a major abiotic stress that limits the production of most crops. Mung bean is mainly distributed in Asia where rainfall is concentrated in the summer, however, drought stress at the seedling stage affects mung bean development [36]. This study explores the inner mechanism of drought response in mung bean at the physiological and molecular levels, which has practical significance.

### Effects of drought stress on mung bean after treated 3 days

Stomatal conductance is regulated by abscisic acid (ABA) accumulation, and is also associated with drought stress by controlling photosynthesis [37]. After 3 days of dehydration (G0), the stomatal conductance of drought-treated samples significantly decreased to 0.16 mol H_2_O m^-2^s^-1^, and the chlorophyl content also significantly decreased (Fig 1B). DEGs related to chlorophyll were negatively correlated with the green module (Fig 5), which refers to carotenoid biosynthesis and chlorophyll metabolism. Chlorophyll is frequently used to assess photosynthetic capacity [38]. When mung bean suffered drought stress for 3 days, the chlorophyll metabolism quickly responded, resulting in weak photosynthesis. As a product of lipid peroxidation, MDA is harmful to plant cells, and high levels of POD and soluble protein can help plants to defend against adverse conditions [39]. The samples of G0 stage, POD decreased significantly (Fig 1D), and more MDA and soluble protein accumulated (Fig 1C), while DEGs involved in signaling transduction became active (Table 3). At the initial stages of drought treatment, mung bean response firstly by increasing signaling transduction, after which downstream genes exert their functions, and plant hormones plays a pivotal role. This contradiction in physical indexes highlights the complexity of the drought response mechanism in mung bean plants [40,41].

### Response mechanism of mung bean to moderate drought stress

Relative water content quickly decreased after drought stress treatment [42]. In this study, when the relative water content of the treated mung bean significantly decreased than that of the control (S1 Appendix), the chlorophyll content, MDA content, POD content, and soluble protein content significantly increased (Fig 1B–1E). The number of DEGs at the G1 stage increased sharply, and DEGs were mainly related to membrane metabolism and gene expression regulation, while down-regulated genes were involved in membrane metabolism, protein kinase activity, oxidoreductase activity, hydrolase activity, plant hormone signal transduction, and photosynthesis (Tables 2 and 3). When the water deficiency become more severe, the gene expression become active and the plant cell membrane metabolism strengthened. With higher soluble protein contents and enhanced POD activity, plant cells adjusted their osmotic potential and eliminated toxicant metabolites to respond to osmotic stress [43].

### Response mechanism of mung bean to withering

As the drought severity intensified, the leaves of drought-treated samples present a withering phenotype, the POD and MDA contents peaked and were higher than that of the control, and the chlorophyll content was not different between the treated samples and the control (Fig 1B–1E). Photosynthesis rapidly responded to drought stress, and as it intensified, the expression of genes related to photosynthesis was down-regulated. resulting in a lower chlorophyll content compared with the G1 stage. The metabolism of plant hormones became active at the G2 and G1 stages (Table 3). Plant hormones play important roles in response to drought stress [44], which highlights the importance of hormones in regulating plant development. The metabolism of starch and sugar affects osmotic stress in plants [45], and in our results, genes related to starch and sugar metabolism were differentially expressed between the control and the treated samples. This indicates that osmotic adjustment is an important component of plant response to drought stress.

### The influence on drought-treated mung bean after of 24 hours of rehydration

Rehydration can reduce drought stress. After 24 h of rehydration (G3), the chlorophyll content increased but was not different from that of the control samples (Fig 1B). The MDA and POD contents in the treated samples were higher than that in the control, but the MDA in treated samples decreased compared with the G2 stage, which emphasized the role of MDA in response to drought stress [46]. The number of DEGs at the G3 stage decreased to 725 (Fig 3), and are mainly related to integral components of membrane and ATP activity (Table 2). There were 4 co-expression DEGs at the G3 stage and the G0 stage, which were mainly involved in abscisic acid and oxidoreductase activity, highlighting the importance of abscisic acid and osmotic adjustment.

## Conclusions

Mung bean is a drought-resistant plant that has complicated response mechanisms to drought stress. In this paper, the mung bean plants showed different characteristics at different levels to water deficit. At the morphological level, the leaves wilted but recovered after rehydration, while drought treatment leads to a dwarf phenotype. At the physiological level, as drought stress intensified, superoxide continually accumulated in cells, and peroxidase was quickly produced to reduce the damage caused by superoxide. Chlorophyll quickly responded to drought stress, and mild stress facilitates the accumulation of chlorophyll, but under severe drought conditions, chlorophyll metabolism decreased. At the molecular level, many genes responded to drought stress. Of these, genes related to DNA binding, membrane metabolism, and protein binding were differentially expressed in all 4 stages, and genes involved in plant hormone and sugar metabolism were expressed actively at the G2 and G3 stages, many genes related to oxidoreductase activity were up-regulated under severe drought stress, while other genes also participated in complex gene expression networks. The RNA-seq results and morphological and physiological indicators outlined in this paper lay a foundation for breeding drought-resistant mung bean cultivars.

## Supporting information

S1 Appendix Data for photosynthetic indexes and relative water content at G0 ang G1 stage. (XLSX)

S2 Appendix Alignment between sample sequenced data and selected reference genome. (XLSX)

S3 Appendix GO enrichment of DEGs. (XLSX)

S4 Appendix DEGs in KEGG pathway. (XLSX)

S5 Appendix TF classify of DEGs. (XLSX)

S6 Appendix Genes in different modules. (XLSX)

S7 Appendix Raw data for Fig 1. (XLSX)

S8 Appendix Raw data for Fig 2. (XLSX)

S9 Appendix Raw data for Fig 3. (XLSX)

S10 Appendix Raw data for Fig 6. (XLSX)
